# Effects of muscle-tendon mechanical properties and electromyographic activity patterns on individual differences in force-power relationship

**DOI:** 10.1371/journal.pone.0350202

**Published:** 2026-05-29

**Authors:** Takehiro Kosaka, Keitaro Kubo

**Affiliations:** 1 Graduate School of Integrated Science and Technology, Nagasaki University, Nagasaki, Japan; 2 Department of Life Science, The University of Tokyo, Meguro, Tokyo, Japan; Padre Anchieta University Center, BRAZIL

## Abstract

This study aimed to examine the effects of muscle-tendon mechanical properties and electromyographic activity patterns on individual differences in the force-power relationship during jumping with and without countermovement. Twenty men executed unilateral jumps using only ankle joint under the following conditions: no-countermovement jump (noCMJ) and countermovement jump (CMJ) with five different loads (0, 10, 30, 50, and 70% of 1 repetition maximum (RM)). During concentric phase of each jump, mean power and electromyographic activities were measured. In addition, the power ratio of higher load conditions (50% and 70% 1RM) to lower load conditions (0% and 10% 1RM) was calculated as an indicator of individual differences in the force-power relationship. Active muscle stiffness of medial gastrocnemius muscle was calculated according to changes in estimated muscle force and fascicle length during fast stretching at three different angular velocities (100, 300, and 500 deg·s^-1^) after submaximal isometric contractions. Tendon stiffness was measured during ramp and ballistic contractions. For noCMJ and CMJ, active muscle stiffness at all angular velocities and the ratios of electromyographic activities were not significantly correlated with the power ratio. Tendon stiffness measured during ramp and ballistic contractions was significantly correlated with the power ratio for noCMJ, but not CMJ. In conclusion, individual differences in the force-power relationship during jumping without countermovement are associated with the tendon mechanical properties, whereas those with countermovement are not related to the muscle-tendon mechanical properties and electromyographic activity patterns.

## Introduction

Force-velocity relationships have been studied at various levels from single muscle fiber to human joint movement, and maximal power (product of force and velocity) is known to occur at around 30–40% of maximum muscle force [1–6]. In sports competitions, the required power is thought to differ across events. For example, power under high-load conditions would be necessary in judo and weightlifting, whereas power under low-load conditions would be necessary in boxing and track and field (especially sprinting and jumping). Although this is not surprising, little attention has been paid to individual differences in the force–power relationship. In general, individual differences in the force-power relationship are thought to be related to muscle-tendon mechanical properties and muscle activity patterns.

Regarding muscle-tendon mechanical properties, they may be strongly related to individual differences in the force-power relationship, especially in jump with countermovement, since several studies have shown a relationship between stretch-shortening cycle performance and muscle-tendon mechanical properties [7–12]. According to previous studies [7,9–12], the lower the tendon stiffness or the higher the muscle stiffness, the higher the performance in stretch-shortening cycle exercises. Based on these findings, it is hypothesized that during the eccentric phase of the stretch-shortening cycle, higher tendon extensibility coupled with lower muscle extensibility enables greater storage of elastic energy in the tendon, thereby facilitating greater power output during the subsequent concentric phase. Furthermore, several studies reported that the strain rate affected the mechanical properties of muscles and tendons [13–16]. In countermovement jump, individuals with low tendon stiffness under low strain rate conditions (i.e., ramp conditions) would have higher exerted power under high load conditions (i.e., low strain rate of tendon), whereas individuals with low tendon stiffness under high strain rate conditions (i.e., ballistic conditions) would have higher exerted power under low load conditions (i.e., high strain rate of tendon). Concerning muscle properties, individuals with higher muscle stiffness (i.e., more tendon stretch) at low angular velocity would have higher exerted power at high load, whereas those with higher muscle stiffness (i.e., more tendon stretch) at high angular velocity would have higher exerted power at low load.

Regarding muscle activity patterns, previous studies demonstrated that the velocity-specific changes due to plyometric training with different loads were associated with changes in muscle electrical activity [17–19]. McBride et al. [19] reported that the increase in electromyographic activities during jumping in the load condition where the training was performed was more pronounced than during jumping in the other load conditions. Thus, if individual differences in the force-power relationship are related to muscle activity patterns, the electromyographic activity would be higher under load conditions with higher exerted power. Furthermore, the influence of electromyographic patterns on individual differences in the force-power relationship in jumps without countermovement is expected to be great because the impact of the above muscle-tendon mechanical properties is expected to be less in jumps without countermovement than in jumps with countermovement.

The purpose of this study was to examine the effects of the mechanical properties of muscles and tendons and electromyographic activity patterns on individual differences in the force-power relationship during jumping with and without countermovement using only ankle joint. We hypothesized that the force-power relationship in no- countermovement jump would be associated with electromyographic activity patterns, whereas the force-power relationship in countermovement jump would be related to muscle-tendon mechanical properties.

## Materials and methods

### Participants

The sample size was estimated using the data from previous studies [9,20,21], in which the effects of muscle-tendon mechanical properties on muscle functions (e.g., joint stiffness and rate of torque development) was determined. Based on an 𝛼 level of 0.05 and a power (1 – *β*) of 0.8, it was shown that at least nineteen participants were necessary for this study. Twenty men (age: 24.5 ± 1.8 yrs, height: 175.0 ± 4.7 cm, body mass: 69.6 ± 8.5 kg, mean ± SD) volunteered to participate in this study (recruitment period: 2023/4/10–2023/7/30). They were physically active, but they hadn’t engaged in resistance training for a minimum of a year before the experiment. They were briefed on the goals of the investigation and the methods that would be employed. Prior to starting the study, written informed consent was acquired. This study conformed to the Declaration of Helsinki and was approved by the Ethics Committee for Human Experiments, Department of Life Science (Sports Sciences), The University of Tokyo (Issue Number: 882). Some of the data in this study (mean torque, angular velocity, power, and electromyographic activities during the concentric phase of jumps for 0%, 30%, and 70% of one repetition maximum (RM)) were presented previously [22].

### Force-power relationship during jumping

Participants performed unilateral jumps with only their right ankle joint on a specifically designed sledge device (AO-3000K, Applied Office, Japan) under the following conditions: no-countermovement jump (noCMJ) and countermovement jump (CMJ) with five different loads (0, 10, 30, 50, and 70% of one repetition maximum (1RM)). At least one week before the measurements, the unilateral 1RM of the plantar flexor muscles was measured in all participants according to a procedure that has been previously described [23]. During jumping test, they were recorded with a sampling rate of 250 Hz using a digital high-speed camera (VCC-H1600C, Digimo, Tokyo, Japan). Four retroreflective markers were attached on the right side of each participant: the tip of the trochanter major, lateral epicondyle of the knee, lateral malleolus, and fifth metatarsophalangeal joint. The vertical reaction force on the force plate (Kistler, 9281B, Switzerland) attached to the platform of the sledge equipment was simultaneously recorded during jumping.

Prior to this measurement, they performed several submaximal jumps in order to acquaint themselves with the test’s protocols. They were instructed to jump as high as they could during each test. For noCMJ, they applied plantar flexion torque after maintaining maximum dorsiflexion until the toe lifted off the surface of the force plate. For CMJ, they kept in the maximal plantarflexed position, then exerted plantar flexion torque to maximal dorsiflexion and immediately rebounded to begin plantar flexion until the toe lifted off the surface of the force plate.

The test was repeated two times per condition with at least 1 min between tests. The motion analysis software (Frame-DIAS ver. 5, DKH, Tokyo, Japan) was used to analyze the ankle joint angle. A fourth order Butterworth-type low-pass filter with a 15 Hz cutoff frequency was used to filter the ankle joint angle data. The ankle joint torque during jumping was estimated from the following equation [24–26]:


$$\begin{array}{c} \text{Ankle joint torque}=\text{Fz}\cdot \text{L}\cdot \text{cos }({\text{A}}_{\text{J}}) \end{array}$$

where Fz, L, and A_J_ represent the vertical reaction force, the length from the center of the ankle joint to ball of the foot, and ankle joint angle. The duration, mean torque, mean angular velocity, and mean power during concentric phase were calculated. The mean values of two trials were used for the following analyses. In the present study, the power ratio of higher load conditions (50% and 70% 1RM) to lower load conditions (0% and 10% 1RM) was calculated as an indicator of individual differences in the force-power relationship. The repeatability of measurement of mean power during jumping was investigated on 2 separate days in a preliminary study with ten males. Regarding noCMJ, the coefficients of variation were 8.5% for 0%, 10.6% for 10%, 6.2% for 30%, 11.4% for 50%, and 10.7% for 70%, respectively. Regarding CMJ, the coefficients of variation were 13.1% for 0%, 8.0% for 10%, 9.7% for 30%, 9.4% for 50%, and 12.7% for 70%, respectively. Regarding power ratio, the coefficients of variation were 11.2% for noCMJ and 12.1% for CMJ, respectively.

Electromyographic activities (EMG) of the lateral gastrocnemius muscle (LG) and soleus muscle (SOL) were recorded using a wireless telemetry device (BioLog DL-5500, S&ME, Japan) at a sampling rate of 1 kHz during jumping. The EMG of the medial gastrocnemius muscle (MG) was not measured because the ultrasound probe was fixed over the muscle belly of the MG in the measurement of active muscle stiffness and tendon stiffness (see below). Surface electrodes (DL-510, S&ME, Japan) were affixed to the skin on the muscle belly. Between 20 and 500 Hz, the raw EMG data were band-pass filtered. EMG amplitude was rectified and averaged (mEMG) during concentric phase. In addition, mean mEMG of LG and SOL was calculated as the mEMG of the plantar flexor muscles (PF). Similar to the power ratio (an indicator of individual differences in the force-power relationship), the EMG ratio of the higher load conditions (50% and 70% 1RM) to the lower load conditions (0% and 10% 1RM) was calculated.

### Active muscle stiffness

Active muscle stiffness was measured using a specially designed dynamometer (T.K.K.S-18035, Takei Scientific Instruments Co., Ltd., Niigata, Japan) with the procedure described in our previous studies [14,20]. On the dynamometer bench, participants lay prone, with a customized belt fastening their body. The knee joint was fully extended, and the ankle joint was set at 100 deg (the foot perpendicular to the tibia = 90 deg with angles greater than 90 deg on plantar flexion). Two straps were used to firmly secure the foot to the dynamometer’s footplate. They first performed several submaximal contractions to familiarize themselves with the measurement, and then were instructed to execute twice 3-s maximal voluntary contraction (MVC). During the measurement of active muscle stiffness, the highest MVC value was used to determine the target torque (see below).

Following a 5-min rest, active muscle stiffness at three different angular velocities (100, 300, and 500 deg·s^-1^) was measured. The dynamometer was set up to apply dorsiflexion of 100–80 deg. Using an oscilloscope to visualize the torque applied, the active muscle stiffness was measured three times for each angular velocity at 50% MVC. Participants were asked to keep the same level of effort during fast dorsiflexion. The order of tasks (100, 300, and 500 deg·s^-1^) was randomized in order to avoid any systematic effects. To equalize the analyzed range of motion among the three angular velocities, periods of 140, 60, and 48 ms following the start of stretch were analyzed at 100, 300, and 500 deg·s^-1^ [14,20]. In addition, the measurement at each angular velocity was conducted twice under relaxed conditions (i.e., 0% MVC condition). The mean torque under the relaxed condition was subtracted from the measured torque under the active condition [27]. The average of three tests served as the measured values. The following formula was used to convert the ankle joint torque (TQ) obtained from the dynamometer to muscle force (Fm) [e.g., 25]:


$$\begin{array}{c} \text{Fm}=\text{k}\cdot \text{TQ}\cdot {\text{MA}}^{-\text{1}} \end{array}$$

where k is the relative value of the physiological cross-sectional area of the medial gastrocnemius muscle (MG) among the plantar flexor muscles [28] and MA was obtained by the tendon excursion method during passive ankle rotation (see below; Tendon stiffness), as described in previous studies [29,30].

During the measurement of active muscle stiffness, real-time ultrasonic apparatus (Prosound *α*7, Hitachi Aloka Medical, Tokyo, Japan) was used to measure the fascicle length of MG. In the computer memory of the apparatus, ultrasonic images were stored at 100 Hz at 100 and 300 deg·s^-1^ and 125 Hz at 500 deg·s^-1^ [14]. In order to synchronize the ultrasonic images with the torque and joint angle, an electric signal was superimposed on them. Active muscular stiffness was defined as the slope of muscle force–fascicle length. The repeatability of measurement of active muscle stiffness was confirmed in our previous studies [14,20].

### Tendon stiffness

Tendon stiffness at two distinct strain rates (see below) was measured utilizing the methodology described in our earlier studies [e.g., 25]. Each foot of the dynamometer (custom-made, VINE, Tokyo, Japan) was firmly attached to the footplate using two straps while the participants lay prone on the test bench. The knee joint was fully extended, and the ankle joint was placed at 90 deg. For ramp contraction, participants were instructed to gradually exert torque from relaxation to MVC around 5 s. For ballistic contraction, participants were instructed to exert isometric torque from relaxation to MVC forcefully and rapidly. Ramp and ballistic contraction measurements were made twice with a 1-min rest between tests.

During the measurement of tendon stiffness, ultrasonic images of MG were captured on a videotape at 60 Hz. The ultrasonic image, ankle angle, and torque were synchronized using a timer. Displacement of the fascicle-aponeurosis junction site indicated the tendon elongation. However, during an isometric contraction, an angular joint rotation that occurred in the direction of ankle plantarflexion also contributed to the movement of this point. An electrical goniometer (Penny and Giles, Newport, UK) was attached on the lateral aspect of the ankle in order to monitor ankle joint rotation during the measurements. Additional measurements were performed under passive conditions from 90 to 99 deg of the ankle joint in order to correct the measurements taken for tendon elongation. Displacement of this point under passive conditions was subtracted from the measured tendon elongation under active conditions [e.g., 31]. In addition, each participant’s moment arm length was measured under passive conditions using the tendon excursion method [29,30].

The measured torque by the dynamometer was converted to muscle force with the same procedure as that used to measure active muscle stiffness. In this study, tendon stiffness was defined as the slope of muscle force and tendon elongation above 50% MVC [e.g., 25]. The repeatability of measurement of tendon stiffness was confirmed in our previous studies [25,32].

### Statistical analysis

All data are presented as means ± SD. The normal distribution of the measured variables was confirmed using the Shapiro-Wilk test. A one-way analysis of variance (ANOVA) with repeated measures was used to detect significant effects of load level (%1RM) on the measured variables during jumping. The significance between means was evaluated using the Bonferroni post-hoc test of crucial differences in the case of significant values of F in the ANOVA. To assess the homogeneity of variance in an ANOVA, Mauchly’s sphericity test was utilized. The Greenhouse-Geisser correction was used in cases when the sphericity assumption was violated. For a one-way ANOVA, the partial eta-squared (pη^2^) was used to calculate the effect size. Based on the distribution of the data, the Pearson’s or Spearman’s correlation coefficient was calculated to evaluate the correlations between the measured variables. The level of significance was set at p < 0.05.

## Results

Table 1 shows the measured variables during jumping. There were no differences in the ankle joint angle at the lowest position among the five different loads for noCMJ (p = 0.592, pη^2^ = 0.036) and CMJ (p = 0.423, pη^2^ = 0.049). Duration during concentric phase significantly increased with increasing load for noCMJ (p < 0.001, pη^2^ = 0.748) and CMJ (p < 0.001, pη^2^ = 0.823). Mean torque significantly increased (p < 0.001, pη^2^ = 0.901 for noCMJ; p < 0.001, pη^2^ = 0.807 for CMJ) and mean angular velocity significantly decreased (p < 0.001, pη^2^ = 0.849 for noCMJ; p < 0.001, pη^2^ = 0.837 for CMJ) with increasing load for noCMJ and CMJ. No significant difference in mean power was found among five different loads for noCMJ (p = 0.326, pη^2^ = 0.058) and CMJ (p = 0.094, pη^2^ = 0.124). mEMG significantly increased with increasing load for noCMJ (p = 0.006, pη^2^ = 0.169), but not CMJ (p = 0.179, pη^2^ = 0.078).

**Table 1:** Measured variables during concentric phase of noCMJ and CMJ.

		Mean (sd)				
		0% 1RM	10% 1RM	30% 1RM	50% 1RM	70% 1RM
noCMJ	Ankle angle at the lowest position (deg)	77.1 (6.2)	77.4 (6.8)	77.1 (6.47)	76.6 (7.2)	77.8 (6.1)
	Duration (s)	0.39 (0.06)	0.40 (0.04)	0.47 (0.07) ***###	0.51 (0.08) ***###	0.53 (0.09) ***###$$
	Mean torque (Nm)	110.1 (25.9)	124.3 (27.7) ***	147.4 (34.5) ***###	173.8 (41.6) ***###$$$	196.7 (44.6) ***###$$$&&&
	Mean angular velocity (deg· s^-1^)	110.1 (27.0)	104.4 (23.7)	87.0 (21.1) ***###	75.6 (19.6) ***###$$	66.0 (16.9) ***###$$$&&&
	Mean power (W)	215.4 (85.8)	228.5 (82.9)	227.5 (89.8)	231.6 (917.0)	228.9 (90.1)
	mEMG of PF (mV· s^-1^)	0.08 (0.03)	0.08 (0.03)	0.08 (0.03)	0.08 (0.03)	0.08 (0.03) *#$
CMJ	Ankle angle at the lowest position (deg)	77.9 (8.5)	76.9 (7.5)	77.3 (7.3)	76.6 (7.1)	78.1 (5.8)
	Duration (s)	0.23 (0.03)	0.25 (0.04)	0.29 (0.04) ***###	0.32 (0.04) ***###$$	0.34 (0.05) ***###$$$&
	Mean torque (Nm)	158.0 (44.5)	171.9 (45.8) ***	187.3 (50.3) ***###	206.3 (51.9) ***###$$	225.4 (54.7)***###$$$&&&
	Mean angular velocity (deg· s^-1^)	170.8 (39.0)	164.8 (36.6)	142.0 (33.0) ***###	128.2 (30.5) ***###$$	109.2 (28.1) ***###$$$&&&
	Mean power (W)	491.2 (253.0)	513.0 (248.1)	480.7 (226.7)	477.4 (212.3)	444.5 (198.5)
	mEMG of PF (mV· s^-1^)	0.07 (0.03)	0.08 (0.04)	0.07 (0.03)	0.08 (0.03)	0.08 (0.03)

noCMJ; jump with coutermovement, CMJ jump with countermovement, PF; plantar flexor muscles.

* significant difference from 0% 1RM (*** p < 0.001).

# significant difference from 10% 1RM (### p < 0.001).

$ significant difference from 30% 1RM ($ p < 0.05, $$ p < 0.01, $$$ p < 0.001).

& significant difference from 50% 1RM (& p < 0.05, && p < 0.01, &&& p < 0.001).

**Fig 1:**
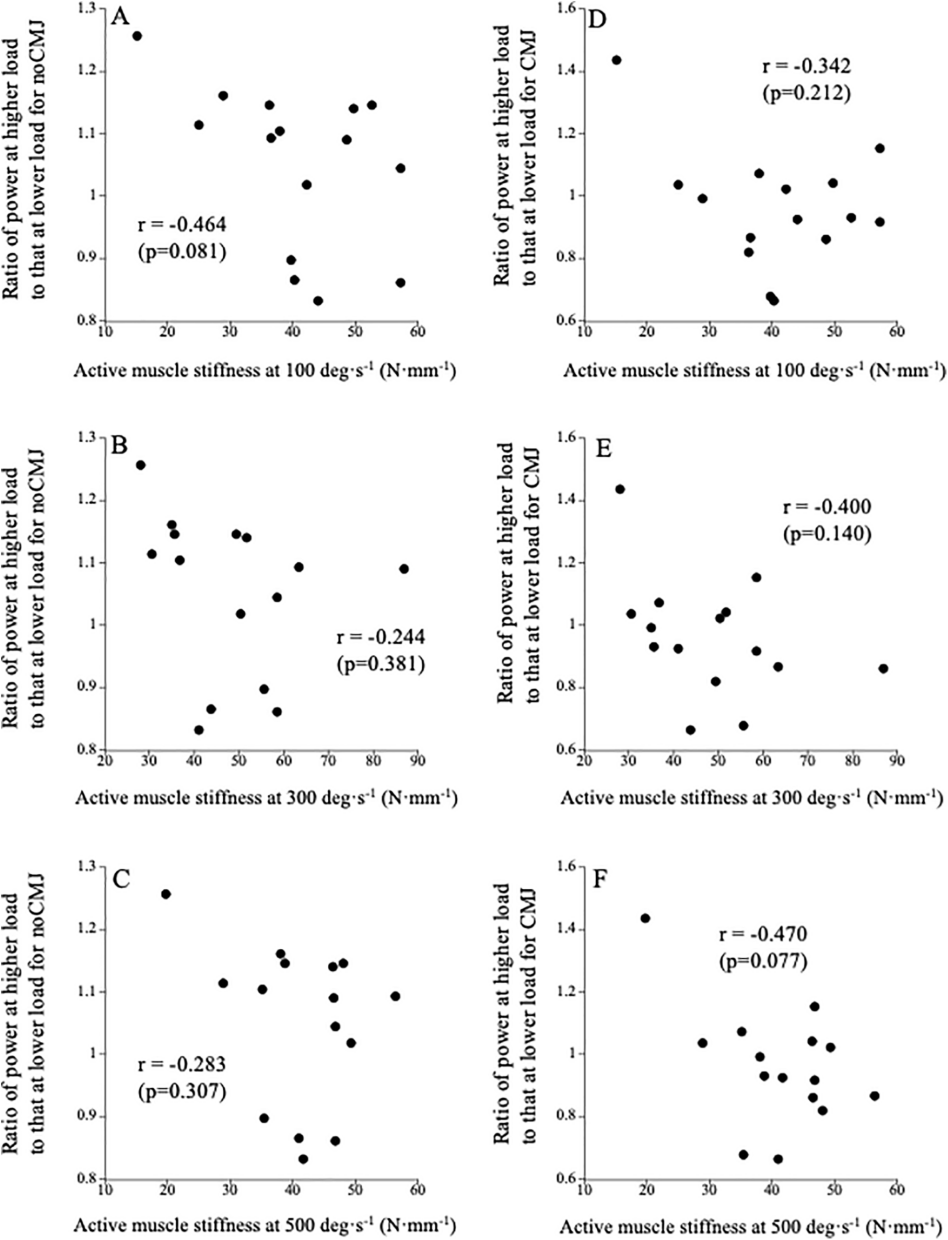
Relationships between active muscle stiffness at 100 (A and D), 300 (B and E), and 500 (C and F) deg·s^-1^ and the ratio of power at higher load to that at lower load for noCMJ (A-C) and CMJ (D-F).

For noCMJ and CMJ, active muscle stiffness at all angular velocities was not significantly correlated with the power ratio (Fig 1).

**Fig 2:**
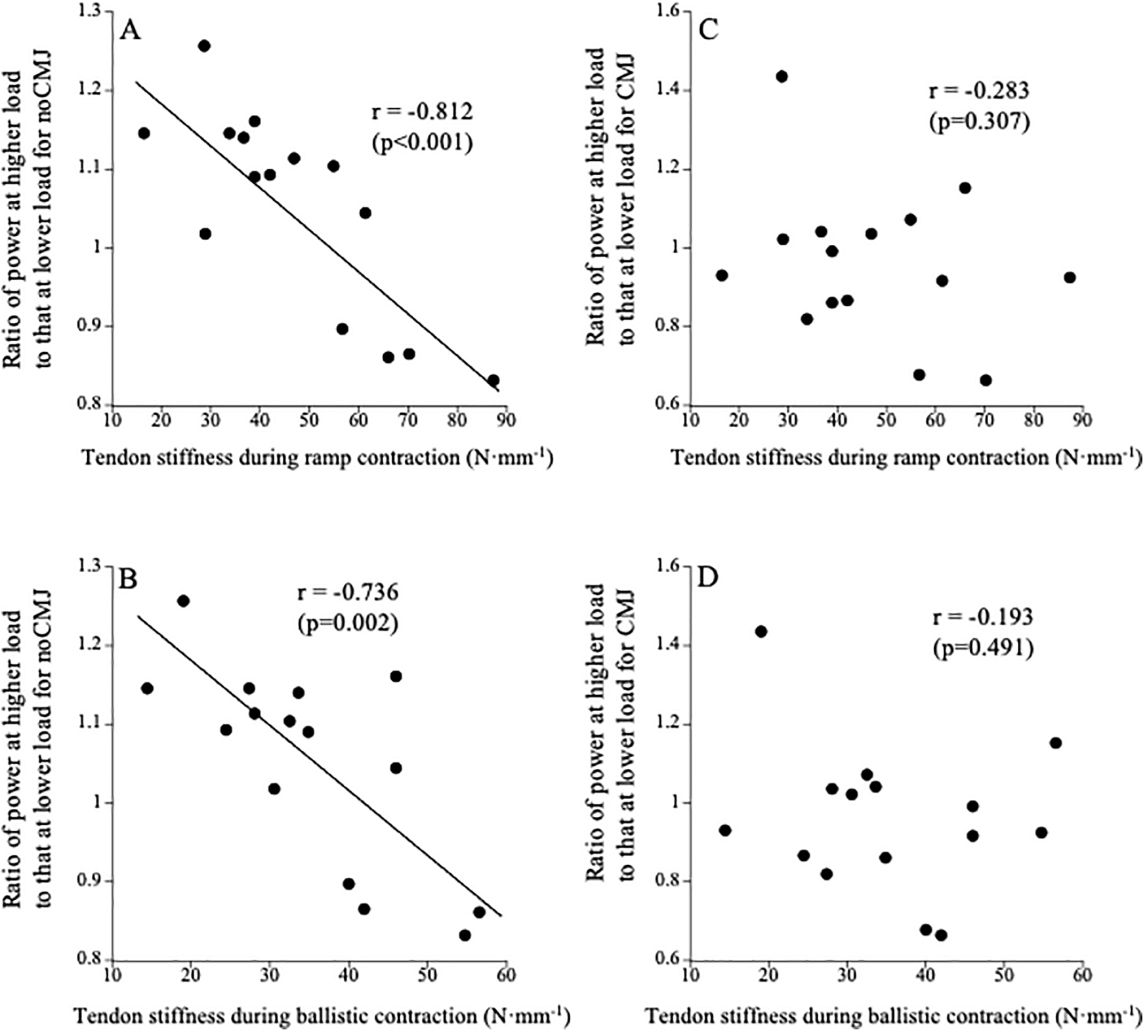
Relationships between tendon stiffness measured during ramp (A and C) and ballistic (B and D) contractions and the ratio of power at higher load to that at lower load for noCMJ (A and B) and CMJ (C and D).

For noCMJ, tendon stiffness measured during ramp and ballistic contractions was significantly correlated with the power ratio (Fig 2A and 2B). For CMJ, however, tendon stiffness measured during ramp and ballistic contractions was not significantly correlated with the power ratio (Fig 2C and 2D).

**Fig 3:**
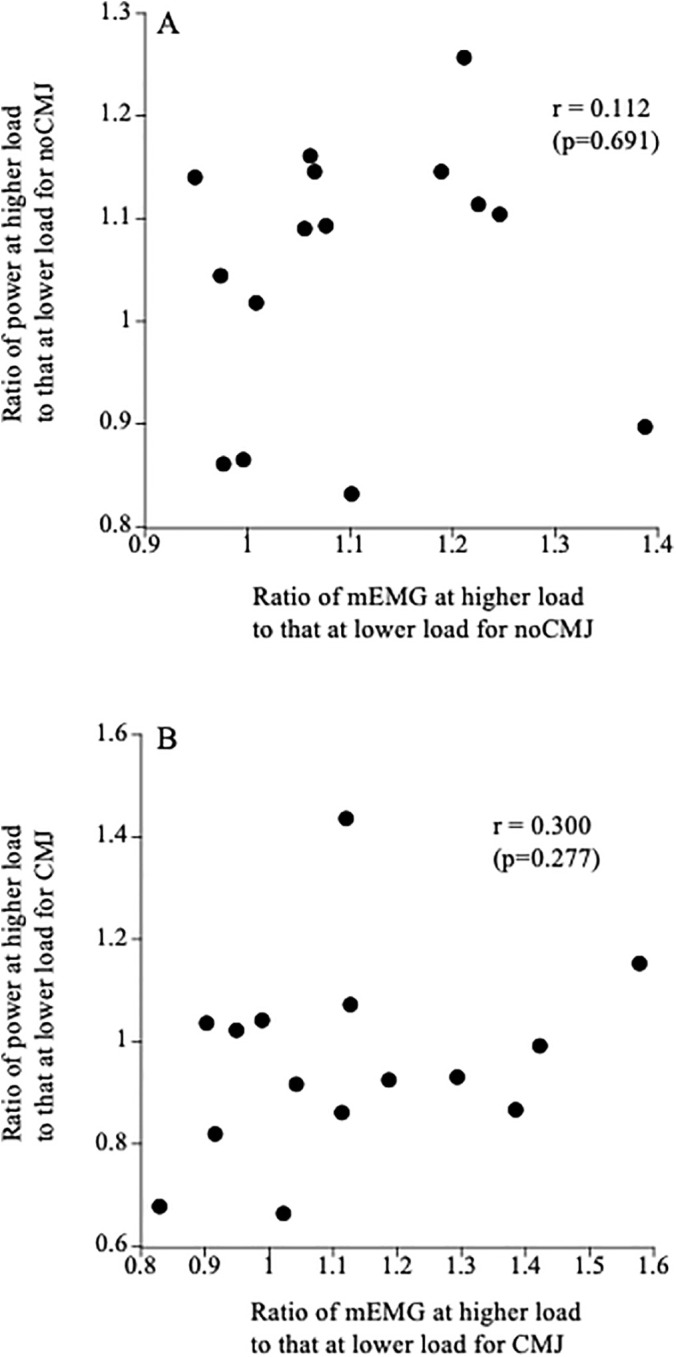
Relationships between the ratio of electromyographic activity at higher load to that at lower load and the ratio of power at higher load to that at lower load for noCMJ (A) and CMJ (B).

For noCMJ and CMJ, the ratio of mEMG at higher load to that at lower load was not significantly correlated with the power ratio (Fig 3).

## Discussion

The force-velocity relationship is known to be hyperbolic, with power peaks appearing at around 30–40% of maximal muscle force [e.g., 6]. However, there are individual differences in the force-velocity-power relationship. In the present study, individual differences in the force-power relationship were evaluated by the ratio of power in the high-load condition (average power in the 50% and 70% 1RM conditions) to power in the low-load condition (average power in the 0% and 10% 1RM conditions). This power ratio was highly correlated with the ratio of combinations among other loads, e.g., power ratio of 50% to 0% 1RM conditions (Table 2). Therefore, the power ratio employed in this study is considered highly valid as an indicator of individual differences in the force-power relationship.

**Table 2:** Correlation coefficient between the power ratio (50%&70%/0%&10%) and the ratio of combinations among other loads.

	noCMJ	CMJ
vs 50%/0%	0.622 **	0.860 ***
vs 50%/10%	0.758 ***	0.816 ***
vs 50%/30%	0.551 *	0.656 **
vs 70%/0%	0.806 ***	0.936 ***
vs 70%/10%	0.777 ***	0.921 ***
vs 70%/30%	0.767 ***	0.916 ***

* p < 0.05, ** p < 0.01, *** p < 0.001.

The power ratios in noCMJ and CMJ were not significantly correlated with active muscle stiffness at all angular velocities (Fig 1). As mentioned in the introduction, we predicted that in jumps with countermovement (i.e., CMJ), which are more closely related to the muscle-tendon mechanical properties, individuals with higher muscle stiffness under high angular velocity conditions would be able to exert higher power during the concentric phase under low load conditions (i.e., lower power ratio) because their tendons would be stretched more during the elongation phase and store more elastic energy. Our recent research also reported that sprinters who regularly train at high velocities (e.g., plyometrics) (who are expected to perform better at even higher velocities) exhibited greater active muscle stiffness at high angular velocities than untrained individuals [33]. However, our hypothesis was rejected in the present study. Unfortunately, the reason for this discrepancy was unclear. In any case, the results of this study indicate that individual differences in the force-power relationship during jumping are not related to active muscle stiffness.

An interesting finding in this study was that the power ratio in noCMJ was negatively correlated with tendon stiffness measured during ramp and ballistic contractions (Fig 2A and 2B). These results implied that those with higher tendon stiffness could exert higher power in the noCMJ under low-load conditions. Unfortunately, we cannot discuss the physiological background behind these results due to lack of data. Since the duration required was shorter under lower load conditions (Table 1), higher tendon stiffness may have favored the exertion of force in shorter periods (i.e., low load conditions). Our previous studies demonstrated that 12 weeks of isometric training increased both rate of torque development and tendon stiffness [34], while three weeks of bed rest decreased both rate of torque development and tendon stiffness [35]. Furthermore, several studies reported that the electromechanical delay was negatively correlated with the Achilles tendon stiffness [36,37]. Accordingly, these results suggested that tendon stiffness was related to force transmission over short periods. In any case, it can be concluded from the results of this study that high tendon stiffness contributes to exerting power in jumping without countermovement under low-load conditions.

Several studies showed that the “velocity specificity” seen in plyometric training with different loads was associated with the changes in electromyographic activities [17–19]. Therefore, in the cross-sectional study (present study), we expected to find a significant correlation between the power ratio and EMG ratio of the high-load condition to the low-load condition. However, this hypothesis was rejected in this study. Therefore, the present result indicated that muscle activity patterns did not affect individual differences in the force-power relationship.

Another interesting result of this study was that no differences in power exerted during jumping using only the ankle joint were found across the different load conditions (Table 1). This result differed from those of previous studies on excised muscle fibers *in vitro* [1,2] and human upper extremity muscles *in vivo* [3,38,39]. In previous studies, maximal power was achieved at 30–40% of maximal muscle strength or at one repetition maximum. On the other hand, previous studies on the human lower extremity muscles have shown inconsistent results on the force-power relationship [40–44]. For example, Cormie et al. [41] and Stone et al. [44] reported that maximum power was seen under minimum load conditions for weighted jumps. Izquierdo et al. [42] demonstrated that the relative load at which maximal power was found during squat by the lower extremity muscle groups (45–60% 1RM) was higher than for bench press by the upper extremity muscle groups (30–45% 1RM). The results of this study indicated that tendon mechanical properties were involved in individual differences in the force-power relationship during jumps performed only with the ankle joint. In particular, the plantar flexor muscles have a long Achilles tendon, and the mechanical properties of the Achilles tendon significantly influence the performance and efficiency of stretch-shortening cycle exercises [45–47]. Therefore, the reason why the results of previous studies on force-power relationships for lower extremity muscle groups do not agree with the results of force-power relationships on excised muscle fibers *in vitro* is that the force-power relationship in human lower-limb muscles may be affected by the tendon mechanical properties of each participant, in addition to the force-power properties of the muscle itself.

There are some limitations to the methodology employed. Firstly, in this study, only two trials of each condition were conducted to minimize the effects of fatigue. The repeatability (coefficient of variation) of measurement of power during jumping (two trials) in each condition for all participants was 9.1% for noCMJ and 10.2% for CMJ on average (see Supporting Information files in S1 and S2 Data). In addition, as described earlier, the repeatability of measurements on different days from a preliminary experiment on 10 participants was also comparable. Therefore, the present study seems to have minimized variation due to fewer trials. Secondly, all participants in this study were untrained individuals. Previous studies reported that the mechanical properties of muscles and tendons in competitive athletes differed from those of untrained individuals [11,47]. Thus, the results of this study may differ from those of the present study if athletes from various disciplines were included in the study. Further research is required to elucidate this topic. Thirdly, this study may have a small sample size. Although we estimated the required sample size based on the results of relevant previous studies [9,20,21], the number of participants in this study may not be considered sufficient. In a future study, the results presented in this study need to be confirmed in a larger sample.

## Conclusions

The present results suggest that while individual differences in the force-power relationship during jumping without countermovement are associated with the tendon mechanical properties, those with countermovement are unrelated to both electromyographic activity patterns and muscle-tendon mechanical properties. Furthermore, the force-power relationship in human plantar flexor muscles differs from that in excised muscle fibers *in vitro*, suggesting that factors other than muscle characteristics are significant modifiers.

## Supporting information

S1 Data Measured variables during noCMJ and CMJ for all participants. (XLSX)

S2 Data Reliability data for the measurement of power ratio. (XLSX)
